# A Protracted Sensitive Period Regulates the Development of Cross-Modal Sound–Shape Associations in Humans

**DOI:** 10.1177/0956797619866625

**Published:** 2019-09-04

**Authors:** Suddha Sourav, Ramesh Kekunnaya, Idris Shareef, Seema Banerjee, Davide Bottari, Brigitte Röder

**Affiliations:** 1 Biological Psychology and Neuropsychology, University of Hamburg; 2 Child Sight Institute, Jasti V. Ramanamma Children’s Eye Care Center, LV Prasad Eye Institute, Hyderabad, India; 3 School of Optometry, The Hong Kong Polytechnic University; 4 IMT School for Advanced Studies Lucca

**Keywords:** sound–shape symbolism, cataract, visual deprivation, cross-modal correspondence, multisensory development, open data, open materials

## Abstract

Humans preferentially match arbitrary words containing higher- and lower-frequency phonemes to angular and smooth shapes, respectively. Here, we investigated the role of visual experience in the development of audiovisual and audiohaptic sound–shape associations (SSAs) using a unique set of five groups: individuals who had suffered a transient period of congenital blindness through congenital bilateral dense cataracts before undergoing cataract-reversal surgeries (CC group), individuals with a history of developmental cataracts (DC group), individuals with congenital permanent blindness (CB group), individuals with late permanent blindness (LB group), and controls with typical sight (TS group). Whereas the TS and LB groups showed highly robust SSAs, the CB, CC, and DC groups did not—in any of the modality combinations tested. These results provide evidence for a protracted sensitive period during which aberrant vision prevents SSA acquisition. Moreover, the finding of a systematic SSA in the LB group demonstrates that representations acquired during the sensitive period are resilient to loss despite dramatically changed experience.

*Cross-modal association* refers to the systematic coupling of a sensory feature in one modality to a sensory feature in another modality. A large body of research has provided evidence that humans systematically associate higher-pitched sounds with angular contours, higher locations in space, and smaller or brighter objects, whereas lower-pitched sounds are preferentially associated with smooth contours, lower locations in space, and larger or darker objects (Brunel, Carvalho, & Goldstone, 2015; Evans & Treisman, 2010; Spence, 2011). Cross-modal associations may not be unique to humans, as suggested by findings indicating that chimpanzees associate higher luminance with higher-pitched sounds (Ludwig, Adachi, & Matsuzawa, 2011).

The correlation between a word’s form (e.g., its phonetic representation) and its meaning can be considered a subcategory of cross-modal associations. In the *bouba-kiki effect*, originally described by Köhler (1929), pseudowords such as *bouba* and *kiki* are preferentially matched to round and angular shapes, respectively. The bouba-kiki effect has been observed nearly universally across cultural and ethnic groups (Chen, Huang, Woods, & Spence, 2016; Köhler, 1929; Oberman & Ramachandran, 2008), with only two known exceptions, both involving languages in which the pseudoword phonemes either are absent or violate phonotactic rules (Rogers & Ross, 1975; Styles & Gawne, 2017). These sound–shape associations (SSAs) have even been reported in the Namibian Himba tribe, which does not have an alphabet (Bremner et al., 2013). Combined with the near universality of the bouba-kiki effect, the latter finding argues against the idea that SSAs originate in any special grapheme–shape association.

Since the discovery of SSAs, the question of whether they are innate or learned has attracted considerable speculation. The near universality of the bouba-kiki effect and the presence of a moderate bouba-kiki effect in toddlers and even prelexical infants have served as evidence that SSAs have an innate basis (Maurer, Pathman, & Mondloch, 2006; Ozturk, Krehm, & Vouloumanos, 2013). For example, Pejovic and Molnar (2017) found evidence that audiovisual SSAs are present by 12 months of age. However, the strongest counterargument for the innateness hypothesis comes from the seminal work of Fryer, Freeman, and Pring (2014), who tested an audiohaptic version of the bouba-kiki effect in blind and partially sighted individuals who matched the pseudowords with haptically perceived shapes. Their findings were contrary to what would be expected if SSAs were innate: Congenitally blind participants in their study did not exhibit any systematic SSAs. A mixed group of late-blind and partially sighted individuals was found performing at an above-chance level, but these participants had a significantly reduced SSA compared with sighted controls.

A recent study in a larger sample of early-blind participants (blindness onset < 2 years of age) and late-blind participants (blindness onset ≥ 3 years of age in the sample) corroborates the claim that SSAs depend on visual experience (Hamilton-Fletcher et al., 2018), although other researchers have suggested special conditions under which SSAs may occur in the early blind (defined as blindness onset < 4 years of age; Bottini, Barilari, & Collignon, 2019). Moreover, no evidence for cross-modal associations between tactile and auditory motions (e.g., a link between increasing pitch and upward motion) has been found in early- and late-blind individuals (defined, respectively, as those with blindness onset ≤ 3 years and after the age of 5 years) but has been observed in sighted individuals (Deroy, Fasiello, Hayward, & Auvray, 2016).

Thus, existing research suggests a crucial role of visual experience in the emergence of some cross-modal correspondences. However, it is still unknown whether there is a sensitive period for cross-modal correspondences, such as the SSA, to be acquired or stabilized. Sensitive periods in development are epochs during which experience has an unusually strong impact on brain functions; after the end of the sensitive period, the acquisition of the same representations is impossible or incomplete (Knudsen, 2004). Determining sensitive phases in typical human functional development requires investigating individuals who suffered a period of blindness at birth but regained vision later. Such individuals allow researchers to determine whether a particular function, such as SSAs, can be acquired after the sensory input that seems to be crucial for its acquisition, such as vision for SSAs (Fryer et al., 2014; Hamilton-Fletcher et al., 2018), becomes belatedly available. In the present study, we tested 30 participants who were born with total bilateral dense congenital cataracts (CC group) and subsequently underwent cataract-removal surgeries. If SSA acquisition depends on a sensitive period in early ontogeny, we would expect a similar pattern of results in an additional group of 15 congenitally permanently blind individuals (CB group)—that is, we would not find the systematic association between sounds and haptic-shapes that we would expect to find in a group of 70 typically sighted control participants (TS group).

Another crucial aspect of sensitive periods is that representations acquired during such periods are not lost (Knudsen, 1998). Thus, individuals who lose their vision after the sensitive period are expected to show systematic sound–haptic-shape associations, as the typically sighted do. This hypothesis was tested in an additional group of 12 late-blind individuals (LB group)—that is, people with blindness onset after the age of 12 years, when multisensory development as assessed in prospective studies comes to or has come to an end (Hillock-Dunn & Wallace, 2012; Nardini, Jones, Bedford, & Braddick, 2008; Röder, Pagel, & Heed, 2013).

Finally, investigating CC individuals allows us to study the recovery of sound–visual-shape correspondence as well. It could be argued that newly gained sight should allow the acquisition of SSAs (or, more generally, the acquisition of cross-modal associations) from the statistics of the natural environment, even if sight becomes available only late. This finding would clearly argue against an early sensitive period for the acquisition of SSAs. Since CC individuals typically still have visual impairments following surgery and recovery, we tested an additional group of 24 individuals with late-onset cataracts after cataract extraction (DC group). DC individuals underwent the same surgical treatment as the other cataract patients and also had some remaining visual impairments. All DC participants tested in the present study had suffered from markedly degraded vision before the age of 12 years. Inclusion of this group allowed us to test whether SSA acquisition is interrupted solely by a phase of congenital total loss of pattern vision or whether later phases of severe visual impairments during childhood also interfere with functional SSAs.

## Method

### Participants

One hundred fifty-four individuals participated in this experiment. Thirty had their vision restored after having total bilateral dense congenital cataracts (CC group; mean age = 18.9 years, range = 6–46 years; 12 female; 28 right-handed; geometric mean visual acuity = 0.207, range = 0.014–0.7, no acuity data for 1 participant; average age at surgery = 58 months, range = 1 month–33 years). Twenty-six had surgery to remove developmental cataracts (DC group). Two participants in this group were rejected because of a history of developmental delays. The 24 remaining DC participants had the following characteristics: mean age = 13.83 years, range = 9–29 years; 13 female; 19 right-handed, 4 with unknown handedness; geometric mean visual acuity = 0.390, range = 0.003–1.00; average age at surgery = 9.30 years, range = 2–17.5 years. All DC individuals had suffered a period of degraded vision before the age of 12 years. We transformed the decimal visual acuities to LogMAR values to meaningfully compare the CC and the DC groups (Holladay, 1997). The DC group had a significantly higher visual acuity than the CC group—one-sided *t* test, *t*(38.877) = 2.064, *p* = .023.

Fifteen participants who lost their vision because of congenital peripheral defects, such as severe forms of Leber’s congenital amaurosis, bilateral congenital anophthalmos, or aggressive retinopathy of prematurity (Stage 5), also took part in the experiment (CB group). One CB participant could discern hand movement at 10 cm; the rest had at most light-projection capacity. Their mean age (also mean blindness duration) was 27.87 years (range: 18–55 years); 6 were female, and 12 were right-handed. Additionally, 13 participants with late permanent blindness were tested for the study (LB group); 1 was excluded for a history of brain tumors and surgery. The remaining 12 had a mean age of 35.33 years (range = 21–61 years) and a mean blindness duration of 9.79 years (range = 6 months–39 years); 5 were female, and 11 were right-handed. Furthermore, 70 TS control participants (mean age = 24.04 years, range = 6–56 years; 52 female; 57 right-handed, handedness data of 6 participants were unknown) took part in the experiment.

All CC, DC, CB, and LB participants were recruited at the LV Prasad Eye Institute, Hyderabad, India, or from the local community of the city of Hamburg, Germany (see Table 1 for distributions of countries of origin and testing). The control participants had normal or corrected-to-normal vision, typical development of all sensory systems, and no neurological disorders. They were recruited from the local community in either Hyderabad, India, or Hamburg, Germany. All visually impaired individuals who participated in the study (CC, DC, CB, and LB participants) were free of any other sensory-system problems and did not have any neurological disorders.

**Table 1.** Participant-Group Abbreviations and Countries of Origin and Testing

Group abbreviation	*n*	Definition	Countries of origin and testing
CC	30	Individuals with a history of total bilateral dense congenital cataracts followed by vision-restoration surgery	27 Indians tested in India, 3 Germans tested in Germany
DC	24	Individuals with a history of developmental cataracts (dense or nondense) before the age of 12 years, followed by cataract-removal surgery	24 Indians tested in India
CB	15	Congenitally permanently blind individuals	9 Indians tested in India; 3 Germans, 1 Russian, 1 Croatian, and 1 Turk tested in Germany
LB	12	Late permanently blind individuals with blindness onset after the age of 12 years	8 Indians tested in India; 2 Germans, 1 Turk, and 1 Briton tested in Germany
TS	70	Typically sighted individuals	28 Indians, 1 Dutch citizen, and 1 Briton tested in India; 31 Germans, 3 Russians, 2 Iranians, 1 Chinese, 1 Kenyan, 1 Kyrgyz, and 1 Japanese tested in Germany

### Ethical approvals

The study was approved in parallel by the institutional ethical review board of LV Prasad Eye Institute, Hyderabad, India, as well as by the local ethical commission of the University of Hamburg Faculty of Psychology and Movement Sciences. The study conformed to the ethical principles of the Declaration of Helsinki (2013).

### Consent and compensation

All participants provided written informed consent for the study. Additionally, the blind participants were orally informed about the general details of the study, data-security policies, and their right to terminate the experiment or withdraw consent for the preservation of collected data at any time. For participants who did not understand English or German, we also orally provided the same information in a language they could fully understand (e.g., Telugu, Hindi, Urdu, Bengali, or Tamil). For minors, a legal custodian’s written informed consent was also obtained. For taking part in the study, adult participants received a small monetary compensation, and the expenses associated with participation (e.g., travel costs) were reimbursed. Minors received a small present instead of monetary compensation.

### Experimental design

#### Stimuli

**Fig. 1.**
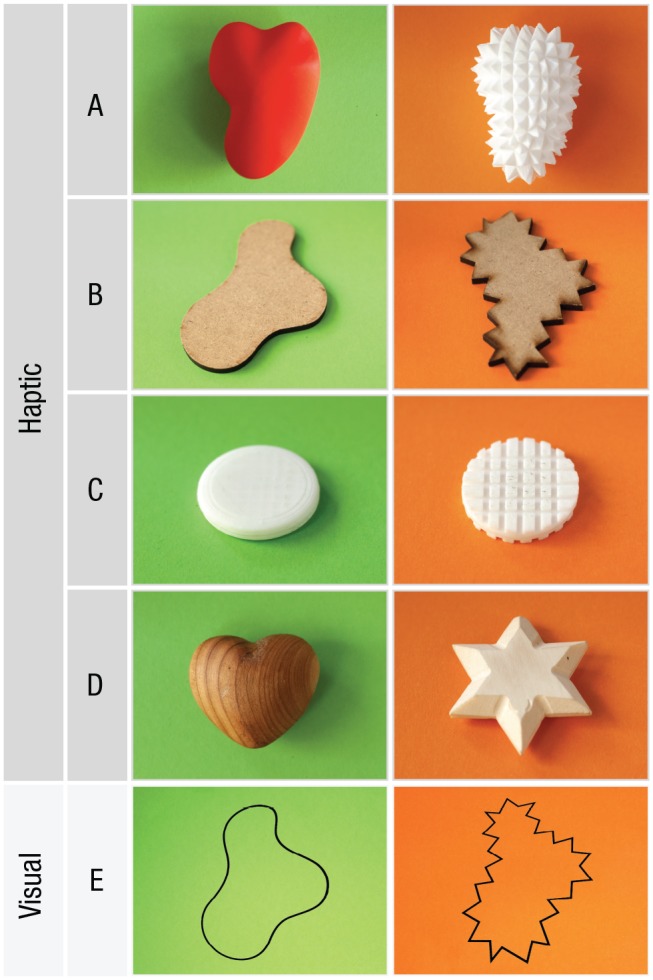
Haptic and visual shape stimuli used in the experiment. Object pairs A, B, C, and D were haptic forms, whereas object pair E was presented on a white background to participants with visual capabilities. Pair A consisted of 3-D models printed in acrylonitrile butadiene styrene polymer. The dimensions of the smallest bounding cuboid were 100 mm × 70 mm × 60 mm. Pair B consisted of flat shapes obtained by laser-cutting plywood. The shapes were about 6 mm thick, and the smallest bounding rectangle dimension was 120 mm × 70 mm. Pair C consisted of 3-D-printed acrylic disks, 40 mm in diameter and 7 mm thick. Pair D consisted of heart and star shapes made of wood, about 70 mm in diameter. Pair E consisted of visually presented shapes printed on white paper. The outlines of the shapes in pair E were exactly the same as those in pair B. Background colors in the figure are for denoting object classes and were not part of the experiment. Object colors of haptic stimuli were not visible to the participants and hence played no role in the task.

We decided to test SSAs rather than other cross-modal correspondences because results from previous studies (Fryer et al., 2014; Hamilton-Fletcher et al., 2018) have most consistently reported deficits for this type of cross-modal correspondence in congenitally blind or early-blind individuals. The set of stimuli consisted of five object pairs, four of which were haptic pairs and one a visual pair (see Fig. 1). Each pair consisted of one object with a smooth shape or texture and another with a spiky shape or texture. The haptic stimuli of pairs A through C closely resembled the three object pairs used in the study of Fryer et al. (2014). Specifically, pair A objects were 3-D printed in acrylonitrile butadiene styrene polymer (Fab Lab, Fabulous St. Pauli, Hamburg, Germany). The smallest bounding cuboid dimension for both objects was 100 mm × 70 mm × 60 mm. Pair B objects were flat shapes laser cut from 6-mm plywood sheets, and the smallest bounding rectangle size was 120 mm × 70 mm. Objects in pair C were 3-D-printed disks with a diameter of 40 mm and a thickness of 7 mm. One of these objects had rounded edges, and the other had a checkerboard-like geometric pattern that imparted a rough texture to the surface. Pair D objects were commercially bought wooden items and had a diameter of approximately 70 mm. Each haptic stimulus pair was presented in a black cloth bag measuring about 45 cm × 30 cm. The outlines comprising pair E were printed side by side on white, A5-size paper and were visually presented. The outlines of the objects in pair E were exactly the same as those of the objects in pair B.

#### Procedure

Separate questionnaires were used for TS participants, participants with a history of cataracts, and permanently blind participants to collect details pertinent to each group. The visual trial was run only in groups with visual capabilities (CC, DC, and TS). For all groups, a precomputed counterbalancing sheet was used to determine the order of trials, with the constraint that the visual trial in the CC, DC, and TS groups was presented either as the first or the last trial.

The experiment was conducted with a script (see Section S1 in the Supplemental Material available online), and instructions were provided in one of the languages the participant was able to understand well (English, German, Telugu, Hindi, Urdu, Bengali, or Tamil). The haptic pairs were handed to each participant one at a time in an opaque black cloth bag closed with a drawstring. Each participant received a haptic object pair exactly once, resulting in four trials involving all four haptic object pairs. Participants were instructed not to look inside the bag but instead to actively explore the contents of the bag by touching them. Thereafter, they were asked to bring out the object matching either bouba or kiki from the bag. The choice of whether to bring out bouba or kiki alternated each trial, and the sequence was counterbalanced across participants in combination with trial order (e.g., trial sequence: CBDAE; retrieval sequence: kiki-bouba-kiki-bouba-kiki; total cycle length: 4! × 2 × 2 = 96 for sighted participants, 4! × 2 = 48 for blind participants). At the end of all trials, participants were asked the reasons dictating their choices. Participants in the CC and the DC groups were also asked to partially copy the shapes of the objects in pair E visually to ensure that they were able to see the outlines in that pair.

#### Response coding

**Fig. 2.**
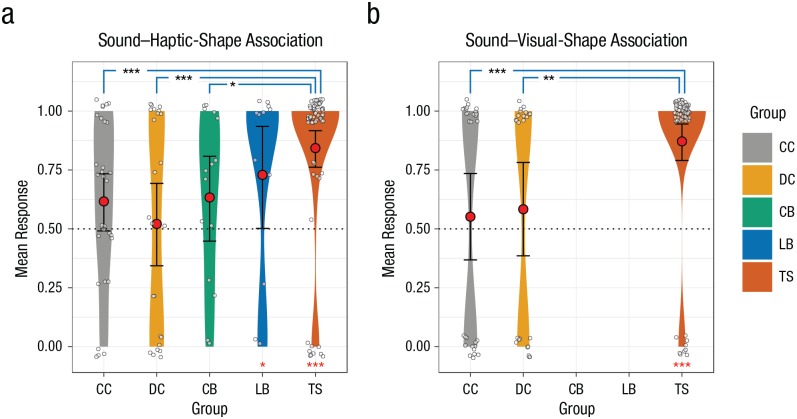
Mean responses in the (a) sound–haptic-shape-association (SSA-H) and (b) sound–visual-shape-association (SSA-V) conditions. Responses are shown separately for individuals with congenital cataracts (CC group), developmental cataracts (DC group), congenital permanent blindness (CB group), late permanent blindness (LB group), and typical sight (TS control group), with kernel density estimated with Gaussian kernels. The width of each plot indicates the density of the data, the red circles indicate group means, the white circles indicate individual data points (jittered for readability), and the error bars indicate 95% confidence intervals of the group means obtained by smoothed bootstrapping with Gaussian kernels. A value of 1 on the *y*-axis indicates a congruent SSA-H or SSA-V (kiki was represented with an angular shape and bouba with a round shape). A value of 0 indicates a incongruent SSA-H or SSA-V (kiki was represented with a round shape and bouba with an angular shape). The dotted line indicates chance-level performance. Only the CC, DC, and TS groups participated in the SSA-V condition. Black asterisks indicate significant differences between groups, and red asterisks indicate significant differences between group mean responses and chance (*p < .05, **p < .01, ***p < .001).

The object brought out or pointed to by the participant was coded on a response sheet. Subsequently, congruent matches were scored as 1 (i.e., kiki matched with an object with an orange background, and bouba matched with an object with a green background; Fig. 1); incongruent matches were scored as 0. In the visual modality, there was only a single trial. For the audiohaptic conditions, the average of the four trial scores was computed for each participant for visualization (see Fig. 2). Therefore, in Figure 2, a score of 1 indicates a completely congruent match in all four trials, and a score of 0 indicates a completely incongruent match. For the statistical analysis, we did not average the binary trials, instead modeling the possibly correlated nature of the trials with a random factor coded by participant ID.

### Statistical analysis

For the three groups with visual capabilities who took part in this task (CC, DC, and TS), we analyzed sound–visual-shape-association (SSA-V) trials using logistic regression models, which implement the maximum-likelihood method, in the R programming environment (Version 3.3.2; R Core Team, 2016). Group (CC, DC, TS) was defined as a categorical factor. Employing two models, we first ascertained whether the probability of congruent SSA-V responses in the CC and the DC groups differed significantly from the probability of congruent SSA-V responses in the TS group (i.e., the difference from the TS group in log odds). Second, we examined whether a systematic SSA-V response was present in each of the groups—that is, whether the log odds of congruent SSA-V responses differed from chance level (zero log odds, *P* = .5) in each group (see Section S2 in the Supplemental Material for a detailed description).

All five groups (CC, DC, CB, LB, and TS) took part in the sound–haptic-shape-association (SSA-H) condition. Because each participant performed four trials, we employed a mixed-effects logistic regression model (Bates, Mächler, Bolker, & Walker, 2015) to test whether any of the visually impaired groups exhibited a statistically significant SSA-H reduction compared with the TS group. In this model, group (CC, DC, CB, LB, TS) was the fixed categorical factor and participant ID served as the random-intercept factor, taking into account the correlated nature of the data in each participant (see Section S2 in the Supplemental Material for a detailed description). Thereafter, we tested whether each group exhibited an SSA-H response that was significantly different from chance level by means of a zero-intercept version of the same mixed-effects logistic regression model. A priori sample-size calculations were performed using simulated data employing the (mixed) logistic regression models (see Section S2 in the Supplemental Material).

## Results

We tested the development and maintenance of cross-modal SSAs in an audiovisual condition (SSA-V) with participants who had recovered their sight (CC group, *n* = 30; DC group, *n* = 24) as well as in a control group of typically sighted participants without any history of visual impairments (TS group, *n* = 70). In the SSA-V condition, participants saw a stimulus pair and had to indicate which shape matched a pseudoword (either bouba or kiki). In the three aforementioned groups and in two additional groups of congenitally, permanently blind participants (CB group, *n* = 15) and late permanently blind participants (LB group, *n* = 12), we ran an SSA-H task using four different haptic-shape pairs. In each of the four trials, participants received a pair of haptic stimuli in an opaque bag and had to indicate which object of the pair matched the pseudoword (bouba or kiki). In the two cataract groups and in the typically sighted group, the audiovisual condition either preceded or followed the audiohaptic conditions in a counterbalanced fashion. In addition, the order of the audiohaptic trials was randomized. The responses were analyzed with generalized linear mixed models (see the Statistical Analysis section, as well as Section S2 in the Supplemental Material).

The audiohaptic responses were analyzed using a mixed-effects logistic regression model with group as the fixed factor. The correlated nature of four trials per participant was modeled by a random-intercept factor for each participant. Comparing the generalized linear mixed models using both a likelihood-ratio test and a parametric bootstrapping test revealed an overall difference in SSA-Hs between groups, χ^2^(4) = 25.846, *p* < .001, parametric bootstrapping: *p* = .001. The consecutive logistic regression model revealed that the CC, the DC, and the CB groups, but not the LB group, significantly differed from the TS group (ΔCC: β = −3.856, 95% confidence interval, or CI = [−6.322, −1.813], *SE* = 1.122, *p* < .001; ΔDC: β = −4.799, 95% CI = [−7.759, −2.463], *SE* = 1.306, *p* < .001; ΔCB: β = −3.621, 95% CI = [−6.722, −0.963], *SE* = 1.420, *p* = .011; ΔLB: β = −1.799, 95% CI = [−5.132, 1.165], *SE* = 1.581, *p* = .255; see Fig. 2). A second analysis to test for the presence of an SSA-H in each of the five groups revealed that only the TS and the LB groups exhibited a significant SSA-H at a higher-than-chance level (TS: β = 5.086, 95% CI = [3.584, 6.881], *SE* = 0.841, *p* < .001; LB: β = 3.287, 95% CI = [0.289, 6.589], *SE* = 1.614, *p* = .042), whereas the CC, the DC, and the CB groups did not perform differently from chance level (CC: β = 1.230, 95% CI = [−0.479, 3.210], *SE* = 0.887, *p* = .165; DC: β = 0.287, 95% CI = [−1.840, 2.510], *SE* = 1.035, *p* = .782; CB: β = 1.465, 95% CI = [−0.989, 4.265], *SE* = 1.265, *p* = .247).

In the audiovisual condition, a similar likelihood-ratio test and parametric bootstrapping test revealed that there was an overall difference between the three groups, χ^2^(2) = 14.808, *p* < .001, parametric bootstrapping: *p* < .001. A logistic regression model revealed that both the CC and the DC groups displayed a significantly reduced SSA-V compared with the TS group (ΔCC: β = −1.706, 95% CI = [−2.751, −0.709], *SE* = 0.517, *p* < .001; ΔDC: β = −1.577, 95% CI = [−2.674, −0.510], *SE* = 0.547, *p* = .004). Moreover, a second model—intended to ascertain the existence of any statistically significant SSA-V in the three groups—revealed that only the TS group, but not the CC group or the DC group, displayed a systematic SSA-V different from chance level (TS: β = 1.914, 95% CI = [1.267, 2.683], *SE* = 0.357, *p* < .001; CC: β = 0.208, 95% CI = [–0.523, 0.957], *SE* = 0.373, *p* = .578; DC: β = 0.337, 95% CI = [−0.468, 1.178], *SE* = 0.414, *p* = .416; see Fig. 2).

## Discussion

In the present study, we investigated the presence of a sensitive period for the development of SSAs. Individuals who regained their sight through vision restoration surgery following a history of a transient congenital or developmental visual impairment due to cataracts were tested in both an audiohaptic (SSA-H) and audiovisual (SSA-V) context, as were sighted control participants. Additionally, congenitally and late permanently blind individuals took part in the SSA-H task.

As predicted by the assumption of a sensitive period in early childhood, we found no evidence for a systematic SSA-H in both the CC and the CB groups. The CB group’s data replicated previous results in similar groups (Fryer et al., 2014; Hamilton-Fletcher et al., 2018), suggesting an essential role of developmental vision for the emergence of SSAs. Crucially, LB individuals showed a significant SSA-H indistinguishable from that of the TS group. This pattern of results demonstrates two remarkable things about sensitive phases: Visual input during childhood development is necessary for the acquisition and stabilization of representations, which seem to be invulnerable to even drastic and long-lasting changes, such as late permanent blindness for up to 39 years. Furthermore, the CC group did not demonstrate an SSA-V either, suggesting that the belatedly available audiovisual–shape statistics were insufficient for SSA acquisition. The absence of SSAs in the audiovisual and audiohaptic domains further supports the notion of ontogenetically early visual input (< 12 years) driving SSA acquisition. The latter is supported and qualified by the findings in the DC group: Like the CC group, the DC group lacked SSAs in both audiovisual and audiohaptic contexts. Since first indications of the bouba-kiki effect have been demonstrated in children and even in infants (Maurer et al., 2006; Ozturk et al., 2013; Pejovic & Molnar, 2017), an intact SSA-H effect in the LB group but not in the DC group suggests that a typical or high visual capability must exist over a protracted developmental phase to elaborate and stabilize cross-modal correspondences such as SSAs; once acquired, these representations seem to be resilient to changing visual environments.

The absence of SSAs in the DC group is remarkable because we have previously demonstrated much higher recovery of extrastriate processing in this group—partially indistinguishable from that of TS individuals—compared with CC individuals (Sourav, Bottari, Kekunnaya, & Röder, 2018). This observation encompasses face processing (Röder, Ley, Shenoy, Kekunnaya, & Bottari, 2013) and visual global motion processing (Bottari et al., 2018) in the DC group. Because all DC individuals suffered from degraded vision before the age of 12 years, the absence of SSAs in this group provides strong evidence for a protracted sensitive period for SSAs before the age of 12 years. Moreover, since all LB individuals in the present study had typical vision until this age, we can conclude that 12 years of intact vision is sufficient for SSA acquisition. Finally, the results of the CC and the DC groups strongly suggest that possible mechanisms of sound–symbolic associations, such as statistical co-occurrence (Sidhu & Pexman, 2018), have sensitive-period constraints, because otherwise both CC and DC groups would have exhibited SSA-Vs driven by extensive exposure to audiovisual statistical properties after sight restoration. Moreover, this account does not explain why the CB group, as well as the CC and DC groups, did not develop normal SSA-Hs.

This pattern of results resembles previous findings in late-blind humans—for example, in the context of spatial reference frames for tactile processing (Collignon, Charbonneau, Lassonde, & Lepore, 2009; Röder, Rösler, & Spence, 2004) and auditory processing (Röder, Kusmierek, Spence, & Schicke, 2007). Late-blind individuals seem to use visual spatial representations despite having suffered partially longer durations of blindness than the CB individuals, who relied in these tasks mostly on body-centered reference frames. Moreover, studies in owls fitted with prisms for a transient phase during the juvenile sensitive period demonstrated that deviant cross-modal spatial associations learned during this period were not lost after prism removal and could be reevoked in adulthood (Knudsen, 1998).

Fryer et al. (2014) reported diminished SSA-Hs in a mixed group of late-blind and partially sighted individuals, and Hamilton-Fletcher et al. (2018) found a lower SSA-H in late-blind participants (blindness onset ≥ 3 years) for low-pitched stimuli. On the basis of our results, we hypothesize that the reported SSA-H reductions might reflect averaging artifacts caused by including late-blind individuals with different histories of visual impairments. Reanalyzing the data of Hamilton-Fletcher et al. (2018) provided evidence for this hypothesis: Including only LB individuals with blindness onset after 12 years of age (*N* = 23), we found a robust SSA-H response that was indistinguishable from the SSA-H of the TS group of the same study (see Section S3 in the Supplemental Material). These findings in the LB group are reminiscent of the higher systematic sound–meaning associations that researchers have observed for words typically learned earlier in language acquisition, with the highest association for words acquired before the age of 13 years (Monaghan, Shillcock, Christiansen, & Kirby, 2014).

It could be argued that the LB individuals had a generally shorter blindness duration compared with that of CB individuals and that a longer blindness duration might have abolished SSA-H effects in this group. Impressively, however, the LB individual with the longest blindness duration (39 years) showed a fully typical SSA-H in our study, as the LB participant in the Hamilton-Fletcher et al. (2018) study did (40 years). Additionally, we found no systematic correlations between SSA-H and blindness duration in LB individuals in the present study or in the study of Hamilton-Fletcher et al. (2018; see Section S3 in the Supplemental Material). Thus, it seems highly unlikely that the blindness duration could account for the difference in SSA-H between the CB and LB individuals.

Although both vision and touch allow shape perception, visual dominance for shape acquisition could be predicted because of the higher spatial resolution afforded by vision, which in turn could foster SSA development and elaboration. If vision, however, does not provide more precise shape information during early ontogeny, SSAs might not be formed or elaborated for visual as well as for haptic shapes. In this context it is remarkable that an emergence of new cross-modal associations has been reported in early-blind individuals (blindness onset < 2 years of age): Unlike sighted participants, early-blind individuals consistently associated higher pitch with smoother or softer textures (Hamilton-Fletcher et al., 2018). Texture can be well perceived by touch, and earlier work has shown that tactile and visual texture information are equally weighted in situations of visual-tactile conflict, unlike in shape conflicts, in which vision dominates (Jones & O’Neil, 1985; Rock & Victor, 1964). Cross-modal correspondence might thus be defined by the dominance pattern of available sensory inputs, which in turn might be defined by the appropriateness of a sensory modality to process certain object aspects (*modality appropriateness*; Welch & Warren, 1980).

Finally, we compared the SSA-V and SSA-H effects of the non-Indian TS subgroup to the Indian TS subgroup and found them to be indistinguishable (see Section S4 in the Supplemental Material), corroborating previous findings that SSA effects emerge independently of cultural backgrounds (Chen et al., 2016; Köhler, 1929; Oberman & Ramachandran, 2008). In the present context, this result excludes cultural differences as a possible alternative explanation for the absence of SSAs in the CC, DC, and CB individuals assessed in India.

In conclusion, the present results suggest that the development and stabilization of audiovisual as well as audiohaptic SSAs in humans depend on high-level vision over a protracted postnatal developmental period. At the same time, we provided evidence that prolonged blindness with an onset after this sensitive period fails to abolish SSAs, demonstrating the other side of the coin of sensitive periods, that is, the robustness of representations acquired during the sensitive period against loss.

## Supplemental Material

Sourav_OpenPracticesDisclosure_new – Supplemental material for A Protracted Sensitive Period Regulates the Development of Cross-Modal Sound–Shape Associations in Humans Click here for additional data file. Supplemental material, Sourav_OpenPracticesDisclosure_new for A Protracted Sensitive Period Regulates the Development of Cross-Modal Sound–Shape Associations in Humans by Suddha Sourav, Ramesh Kekunnaya, Idris Shareef, Seema Banerjee, Davide Bottari and Brigitte Röder in Psychological Science

## Supplemental Material

Sourav_SupplementalMaterial_rev – Supplemental material for A Protracted Sensitive Period Regulates the Development of Cross-Modal Sound–Shape Associations in Humans Click here for additional data file. Supplemental material, Sourav_SupplementalMaterial_rev for A Protracted Sensitive Period Regulates the Development of Cross-Modal Sound–Shape Associations in Humans by Suddha Sourav, Ramesh Kekunnaya, Idris Shareef, Seema Banerjee, Davide Bottari and Brigitte Röder in Psychological Science

## References

[bibr1-0956797619866625] Bates D. Mächler M. Bolker B. Walker S. (2015). Fitting linear mixed-effects models using lme4. Journal of Statistical Software, 67(1). doi:10.18637/jss.v067.i01

[bibr2-0956797619866625] Bottari D. Kekunnaya R. Hense M. Troje N. F. Sourav S. Röder B. (2018). Motion processing after sight restoration: No competition between visual recovery and auditory compensation. NeuroImage, 167, 284–296. 29175496 10.1016/j.neuroimage.2017.11.050

[bibr3-0956797619866625] Bottini R. Barilari M. Collignon O. (2019). Sound symbolism in sighted and blind: The role of vision and orthography in sound-shape correspondences. Cognition, 185, 62–70. 30660923 10.1016/j.cognition.2019.01.006

[bibr4-0956797619866625] Bremner A. J. Caparos S. Davidoff J. de Fockert J. Linnell K. J. Spence C. (2013). “Bouba” and “Kiki” in Namibia? A remote culture make similar shape–sound matches, but different shape–taste matches to Westerners. Cognition, 126, 165–172. 23121711 10.1016/j.cognition.2012.09.007

[bibr5-0956797619866625] Brunel L. Carvalho P. F. Goldstone R. L. (2015). It does belong together: Cross-modal correspondences influence cross-modal integration during perceptual learning. Frontiers in Psychology, 6, Article 358. doi:10.3389/fpsyg.2015.00358 PMC4390988 25914653

[bibr6-0956797619866625] Chen Y.-C. Huang P.-C. Woods A. Spence C. (2016). When “Bouba” equals “Kiki”: Cultural commonalities and cultural differences in sound-shape correspondences. Scientific Reports, 6, Article 26681. doi:10.1038/srep26681 PMC4882484 27230754

[bibr7-0956797619866625] Collignon O. Charbonneau G. Lassonde M. Lepore F. (2009). Early visual deprivation alters multisensory processing in peripersonal space. Neuropsychologia, 47, 3236–3243. 19666035 10.1016/j.neuropsychologia.2009.07.025

[bibr8-0956797619866625] Deroy O. Fasiello I. Hayward V. Auvray M. (2016). Differentiated audio-tactile correspondences in sighted and blind individuals. Journal of Experimental Psychology: Human Perception and Performance, 42, 1204–1214. 26950385 10.1037/xhp0000152

[bibr9-0956797619866625] Evans K. K. Treisman A. (2010). Natural cross-modal mappings between visual and auditory features. Journal of Vision,10(1), Article 6. doi:10.1167/10.1.6 PMC2920420 20143899

[bibr10-0956797619866625] Fryer L. Freeman J. Pring L. (2014). Touching words is not enough: How visual experience influences haptic-auditory associations in the “Bouba-Kiki” effect. Cognition, 132, 164–173. 24809744 10.1016/j.cognition.2014.03.015

[bibr11-0956797619866625] Hamilton-Fletcher G. Pisanski K. Reby D. Stefańczyk M. Ward J. Sorokowska A. (2018). The role of visual experience in the emergence of cross-modal correspondences. Cognition, 175, 114–121. 29502009 10.1016/j.cognition.2018.02.023

[bibr12-0956797619866625] Hillock-Dunn A. Wallace M. T. (2012). Developmental changes in the multisensory temporal binding window persist into adolescence. Developmental Science, 15, 688–696. 22925516 10.1111/j.1467-7687.2012.01171.x PMC4013750

[bibr13-0956797619866625] Holladay J. T. (1997). Proper method for calculating average visual acuity. Journal of Refractive Surgery, 13, 388–391. 9268940 10.3928/1081-597X-19970701-16

[bibr14-0956797619866625] Jones B. O’Neil S. (1985). Combining vision and touch in texture perception. Perception & Psychophysics, 37, 66–72. 3991320 10.3758/bf03207140

[bibr15-0956797619866625] Knudsen E. I. (1998). Capacity for plasticity in the adult owl auditory system expanded by juvenile experience. Science, 279, 1531–1533. 9488651 10.1126/science.279.5356.1531

[bibr16-0956797619866625] Knudsen E. I. (2004). Sensitive periods in the development of the brain and behavior. Journal of Cognitive Neuroscience, 16, 1412–1425. 15509387 10.1162/0898929042304796

[bibr17-0956797619866625] Köhler W. (1929). Gestalt psychology. New York, NY: Liveright.

[bibr18-0956797619866625] Ludwig V. U. Adachi I. Matsuzawa T. (2011). Visuoauditory mappings between high luminance and high pitch are shared by chimpanzees (*Pan troglodytes*) and humans. Proceedings of the National Academy of Sciences, USA, 108, 20661–20665. 10.1073/pnas.1112605108 PMC3251154 22143791

[bibr19-0956797619866625] Maurer D. Pathman T. Mondloch C. J. (2006). The shape of boubas: Sound-shape correspondences in toddlers and adults. Developmental Science, 9, 316–322. 16669803 10.1111/j.1467-7687.2006.00495.x

[bibr20-0956797619866625] Monaghan P. Shillcock R. C. Christiansen M. H. Kirby S. (2014). How arbitrary is language? Philosophical Transactions of the Royal Society B: Biological Sciences, 369(1651), Article 20130299. doi:10.1098/rstb.2013.0299 PMC4123678 25092667

[bibr21-0956797619866625] Nardini M. Jones P. Bedford R. Braddick O. (2008). Development of cue integration in human navigation. Current Biology, 18, 689–693. 18450447 10.1016/j.cub.2008.04.021

[bibr22-0956797619866625] Oberman L. M. Ramachandran V. S. (2008). Preliminary evidence for deficits in multisensory integration in autism spectrum disorders: The mirror neuron hypothesis. Social Neuroscience, 3, 348–355. 18979385 10.1080/17470910701563681

[bibr23-0956797619866625] Ozturk O. Krehm M. Vouloumanos A. (2013). Sound symbolism in infancy: Evidence for sound–shape cross-modal correspondences in 4-month-olds. Journal of Experimental Child Psychology, 114, 173–186. 22960203 10.1016/j.jecp.2012.05.004

[bibr24-0956797619866625] Pejovic J. Molnar M. (2017). The development of spontaneous sound-shape matching in monolingual and bilingual infants during the first year. Developmental Psychology, 53, 581–586. 27854461 10.1037/dev0000237

[bibr25-0956797619866625] R Core Team. (2016). R: A language and environment for statistical computing. Retrieved from https://www.r-project.org/

[bibr26-0956797619866625] Rock I. Victor J. (1964). Vision and touch: An experimentally created conflict between the two senses. Science, 143, 594–596. 14080333 10.1126/science.143.3606.594

[bibr27-0956797619866625] Röder B. Kusmierek A. Spence C. Schicke T. (2007). Developmental vision determines the reference frame for the multisensory control of action. Proceedings of the National Academy of Sciences, USA, 104, 4753–4758. 10.1073/pnas.0607158104 PMC1838672 17360596

[bibr28-0956797619866625] Röder B. Ley P. Shenoy B. H. Kekunnaya R. Bottari D. (2013). Sensitive periods for the functional specialization of the neural system for human face processing. Proceedings of the National Academy of Sciences, USA, 110, 16760–16765. 10.1073/pnas.1309963110 PMC3801039 24019474

[bibr29-0956797619866625] Röder B. Pagel B. Heed T. (2013). The implicit use of spatial information develops later for crossmodal than for intramodal temporal processing. Cognition, 126, 301–306. 23099123 10.1016/j.cognition.2012.09.009

[bibr30-0956797619866625] Röder B. Rösler F. Spence C. (2004). Early vision impairs tactile perception in the blind. Current Biology, 14, 121–124. 14738733

[bibr31-0956797619866625] Rogers S. K. Ross A. S. (1975). A cross-cultural test of the Maluma-Takete phenomenon. Perception, 4, 105–106. 1161435 10.1068/p040105

[bibr32-0956797619866625] Sidhu D. M. Pexman P. M. (2018). Five mechanisms of sound symbolic association. Psychonomic Bulletin & Review, 25, 1619–1643. 28840520 10.3758/s13423-017-1361-1

[bibr33-0956797619866625] Sourav S. Bottari D. Kekunnaya R. Röder B. (2018). Evidence of a retinotopic organization of early visual cortex but impaired extrastriate processing in sight recovery individuals. Journal of Vision, 18(3), Article 22. doi:10.1167/18.3.22 29677338

[bibr34-0956797619866625] Spence C. (2011). Crossmodal correspondences: A tutorial review. Attention, Perception, & Psychophysics, 73, 971–995. 10.3758/s13414-010-0073-7 21264748

[bibr35-0956797619866625] Styles S. J. Gawne L. (2017). When does Maluma/Takete fail? Two key failures and a meta-analysis suggest that phonology and phonotactics matter. i-Perception, 8(4), Article 2041669517724807. doi:10.1177/2041669517724807 PMC5574486 28890777

[bibr36-0956797619866625] Welch R. B. Warren D. H. (1980). Immediate perceptual response to intersensory discrepancy. Psychological Bulletin, 88, 638–667. 7003641

