# Supplementary material for: A Protracted Sensitive Period Regulates the Development of Cross-Modal Sound–Shape Associations in Humans
Source: Psychol Sci. 2019 Sep 4;30(10):1473–82. doi: 10.1177/0956797619866625 (PMC6787766; doi:10.1177/0956797619866625)
Supplement: Sourav_SupplementalMaterial_rev – Supplemental material for A Protracted Sensitive Period Regulates the Development of Cross-Modal Sound–Shape Associations in Humans [file Sourav_SupplementalMaterial_rev.pdf]

Supplemental Online Material

S1. Experiment Script

Audiovisual Trial. On this sheet, you see two shapes. The name of one of the shapes is *Kiki*, and the name of the other shape is *Bouba*.

I want you to point out the object that you think is <Kiki | Bouba>.

Audiohaptic Trials. ↦ [Hands over bag.]

In this bag, there are two shapes. Put one or both of your hands in the bag and feel them without looking inside the bag.

[Pause]

Have you felt both shapes?

[Proceed if the answer is yes, else pause for a few more seconds.]

The name of one of the shapes is *Kiki,* and the name of the other shape is *Bouba*. I want you to bring out the object which you think is <Bouba | Kiki>.

↤

[Repeat three times from ↦ to ↤.]

At the End of the Experiment

Thank you very much! (Non-confrontationally) Why did you choose the shapes this way?

To CC/DC participants: Please copy these shapes for me (point to the objects in pair E).

S2. Detailed Descriptions of Statistical Analyses

Audiovisual Responses. Only the CC, DC, and the TS participants took part in the sound–visual shape association (SSAv) trial. Since this was a Bernoulli trial, to find out whether the CC or the DC group exhibited a statistically significant reduction of SSAv compared to the typically sighted controls, a logistic regression model using maximum likelihood method was employed in *R* v3.3.2 using the generalized linear model function *glm* with a binomial link function (R Core Team, 2016). The single factor *Group* was dummy-

coded such that the TS group served as the reference group and two dummy variables represented the difference in log odds of the CC and the DC group from the TS group (Hardy, 1993). The following equation represents the model employed:

$$\ln\left(\frac{P}{1-P}\right) = \beta_{TS} + \beta_{\Delta CC}X_{\Delta CC} + \beta_{\Delta DC}X_{\Delta DC} + \varepsilon \quad (1)$$

Here β_{TS} is the intercept term, modeling the log odds of a congruent SSAv response for the TS group, and the dummy variables $X_{\Delta CC}$ and $X_{\Delta DC}$ model the difference in log odds of the CC and the DC group respectively from the TS group, allowing us to investigate an SSAv reduction in the cataract groups compared to sighted controls.

The corresponding R equation for the model was *Response ~ 1 + Group*, after setting the TS group as the reference group with the *relevel* function. To check for a significant overall effect of the factor *Group,* we used the parametric bootstrap method using the *PBmodcomp* function with 1000 simulations from the *pbkrtest* v0.4-7 package (Halekoh & Højsgaard, 2014), comparing the model in equation (1) with a null model that fitted an intercept-only model without using *Group* as a factor (*Response ~ 1*). A statistically significant difference between the model in equation (1) and the null model indicates an overall effect of the factor *Group.* In the presence of a significant overall effect of the factor *Group*, a statistically significant coefficient term ($\beta_{\Delta CC}$ or $\beta_{\Delta DC}$) indicate a statistically significant pairwise SSAv difference between the group coded by the dummy variable, and the TS group.

To check for the presence of an SSAv different from chance level ($P = 0.5$) in each of the three groups, another logistic regression model was used. Since we had only one categorical independent variable (*Group*), instead of dummy coding, for this regression the intercept term was forced to zero, and all three groups served as categorical effect terms. Therefore, the modeled equation was:

$$\ln\left(\frac{P}{1-P}\right) = \beta_{TS}X_{TS} + \beta_{CC}X_{CC} + \beta_{DC}X_{DC} + \varepsilon \quad (2)$$

Because a zero log odds $\left(\ln\left(\frac{P}{1-P}\right)\right)$ in this model corresponds to an SSAv probability of 0.5 ($P = 0.5$), that is, a chance level performance, the presence of a statistically significant coefficient term (β_{TS} or β_{CC} or β_{DC}) in this zero-intercept model indicates a systematic SSAv different from chance level for the group corresponding to that term. The R equation for the model was *Response ~ 0 + Group.* As with the first model, to check for a significant overall effect of the variable *Group*, we used the parametric bootstrap method with 1000 simulations by comparing this model with the intercept-only model (*Response ~ 1*).

It should be noted that internally, equations 1 and 2 generate the same model and therefore have exactly the same model parameters, but they test two different a priori hypotheses. The same holds for equations 3 and 4 (see *Results* in main text). We assessed the 95% confidence interval for each of the coefficients using smoothed bootstrap with a Gaussian kernel (Wolodzko, 2018).

Audiohaptic Responses. Compared to the analysis of the sound–visual shape condition, there were two differences in the analysis of sound–haptic shape condition (SSAh) data. First, participants of all five groups (TS, CC, DC, CB, and LB) took part in the latter condition. Second, each participant performed 4 trials. Our model accommodated this organization of the data as outlined below.

We employed a mixed effects logistic regression model applying the maximum likelihood method using the *lme4* v1.1-17 package (Bates, Mächler, Bolker, & Walker, 2015) to test whether any of the visually impaired groups exhibited a statistically significant SSAh reduction compared to typically sighted controls. The fixed factor *Group* was dummy-coded as in the audiovisual analysis with the TS group serving as the reference group, and four dummy variables representing the difference of log odds of the CC, DC, CB and LB group

respectively from the TS group. As each participant contributed to 4 trials, we used *Participant ID* as a random intercept factor to take the possibly correlated nature of the data into account. The logistic regression modeled the following equation in R:

$$\ln\left(\frac{P}{1-P}\right) = \beta_{TS} + \beta_{\Delta CC}X_{\Delta CC} + \beta_{\Delta DC}X_{\Delta DC} + \beta_{\Delta CB}X_{\Delta CB} + \beta_{\Delta LB}X_{\Delta LB} + u_i + \varepsilon \quad (3)$$

Where u_i is the ith subject-specific random intercept term. The corresponding R equation was *Response ~ 1 + Group + (1|ParticipantID)*. To check for a significant overall effect of the fixed factor *Group,* we used parametric bootstrap using the *PBmodcomp* function with 1000 simulations from the *pbkrtest* package (version 0.4-7) comparing this model to a null model that did not contain the fixed effect term (*Response ~ 1 + (1|ParticipantID)*). As with equation (1), in the presence of an overall effect of *Group*, each statistically significant coefficient term in equation (3) ($\beta_{\Delta CC}/\beta_{\Delta DC}/\beta_{\Delta CB}/\beta_{\Delta LB}$) indicates a statistically significant pairwise SSAh difference between the group coded by the dummy variable, and the TS group.

A second mixed-effect logistic regression model was used to check for the presence of a statistically significant SSAh different from chance level ($P = 0.5$) in the five groups. As in equation (2), instead of dummy coding, for this model the intercept term was forced to zero and all five groups served as categorical fixed effect terms. Therefore, the modeled equation was:

$$\ln\left(\frac{P}{1-P}\right) = \beta_{TS}X_{TS} + \beta_{CC}X_{CC} + \beta_{DC}X_{DC} + \beta_{CB}X_{CB} + \beta_{LB}X_{LB} + u_i + \varepsilon \quad (4)$$

Analgous to equation (2), a log odds of zero in this model corresponds to $P = 0.5$, that is, a chance level performance. The presence of a statistically significant coefficient term for any of the variables in this zero-intercept model ($\beta_{TS}/\beta_{CC}/\beta_{DC}/\beta_{CB}/\beta_{LB}$) indicates a systematic SSAh different from chance level in the corresponding group. The R equation for this model was *Response ~ 0 + Group + (1|ParticipantID)*. As in the first model, we used the parametric

bootstrap method with 1000 simulations (Halekoh & Højsgaard, 2014) for ascertaining a statistically significant overall effect of *Group* comparing it to the null model *Response ~ 1 + (1|ParticipantID)*. Smoothed bootstrap with a Gaussian kernel was used to calculate the 95% confidence interval of the regression coefficients (Wolodzko, 2018).

Sample Size Determination. There was a single trial in the audiovisual condition whereas the audiohaptic condition consisted of four trials. The audiovisual condition, being lower-powered, allowed us to assess a minimum sample size for the groups with visual capabilities (CC, DC, and TS). To assess sample size, we simulated 1000 datasets with three groups resembling the CC, DC, and the TS group. A-priori, we assigned the probabilities 0.65, 0.74 (corresponding to a Cohen's *h* of 0.5, that is, a medium effect size for departure from chance level), and 0.8 for the three groups respectively and generated binomial trials with these probabilities. The sample size of the three groups was varied from 5 to 80 in steps of 5 and thereupon we performed the same analysis as mentioned in *Statistical Analysis of Audiovisual Responses.* The achieved power for a medium effect size is plotted against the sample size in Fig. S2.1, indicating a sample size of about 30 in the audiovisual condition per group. Although we aimed for 30 participants in both CC and the DC groups, due to availability of participants and pre-existing conditions we had 24 DC participants entering the analysis (Power ≈ 0.7).

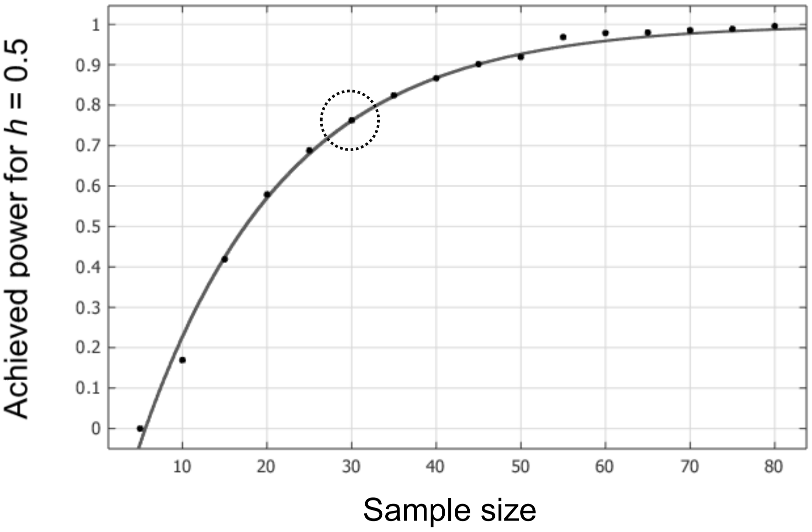

***Figure S2.1*. Sample size determination for the groups with visual capabilities.** Achieved power for a Cohen's h = 0.5 plotted against the sample size of the groups for the audiovisual analysis using simulated data (N = 1000 per group per data point). The solid line is the best fit line in the form of an exponential subtracted from 1, that is, $1 - exp(ax - b)$.

In the audiohaptic condition, there were four trials per participant, providing a higher power. Therefore, the participants who only took part in audiohaptic trials (CB and LB groups) could be tested with a smaller sample size. Similar to the previous section, we generated 1000 datasets for the five groups (CC, DC, CB, LB, and TS) varying the sample size from 5 to 80 in steps of 5. Trials were generated by an independent random binomial trial generator with the probabilities of 0.5, 0.6, 0.7, 0.74 (Cohen's h = 0.5, indicating a medium effect size), and 0.8 for the five groups in order.

The achieved power for this analysis is plotted in Fig. S2.2, indicating that with a minimum sample size of 10 per group we achieve a power that is greater than 0.8 in this case. We used a sample size of 70 for the TS group in the present study to allow us to detect possible group differences from a typical SSA pattern observed in TS individuals with a higher power.

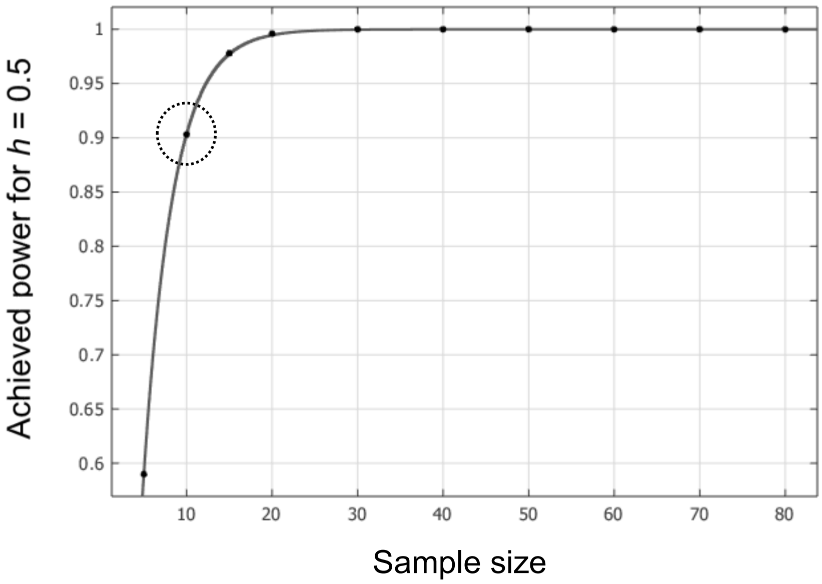

***Figure S2.2*. Sample size determination for the blind groups.** Achieved power for a Cohen's $h = 0.5$ plotted against the sample size of the group for the audiohaptic analysis using simulated data ($N = 1000$ per group per data point). The solid line is the best fit line in the form of an exponential subtracted from 1, that is, $1 - exp(ax - b)$. From N = 20 onwards, data points in intervals of 10 samples are shown.

S3. Reanalysis of the SSA Data of Hamilton-Fletcher et al. (2018) and Effect of Blindness Duration on SSAh in LB Individuals

We reanalyzed the SSAh data of Hamilton-Fletcher et al. (2018) using two models similar to the SSAh regression models employed in the present study (see *S2. Detailed Descriptions of Statistical Analyses: Audiohaptic Responses*). The only difference was the presence of three groups (Early blind, EB; late blind, LB; and typically sighted controls, TS) instead of five groups as in our study.

Hamilton-Fletcher et al. (2018) defined early blindness as blindness onset before the age of two years. The LB participants in their study included persons whose blindness onsets were at 3 years of age or later. When we used this criterion for late blindness (onset of blindness ≥ 3 years, $N = 27$), we obtained evidence for a statistically significantly lower SSAh compared to sighted controls ($N_{TS} = 63$, $N_{LB} = 27$, $\beta_{\Delta \mathrm{LB}} = -2.506$, $SE = 1.106$, $p = 0.023$, 95% CI [-4.982, -0.471]). However, this statistically significant difference from the TS group vanished when only LB individuals with blindness onsets after the age of 12 were included, as in the present study ($N_{LB} = 23$, $\beta_{\Delta \mathrm{LB}} = -1.874$, $SE = 1.237$, $p = 0.130$, 95% CI [-4.581,

0.487], see Fig. S3). The EB group, on the other hand, was found to have a statistically significantly lower SSAh compared to the TS group ($N = 32$, $\beta_{\Delta EB} = -4.230$, $SE = 1.259$, $p < 0.001$, 95% *CI* [-7.241, -2.095], see Fig. S3).

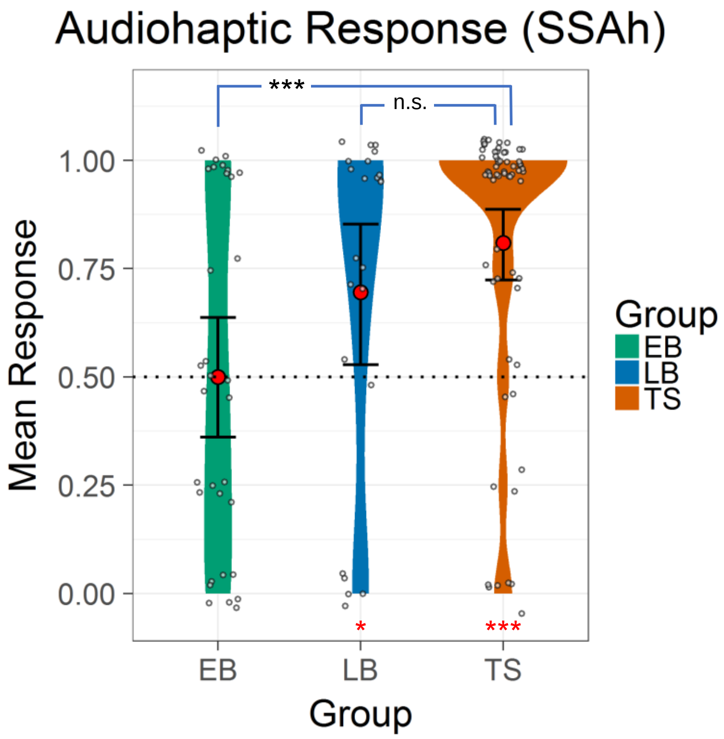

***Figure S3*. Reanalysis of the data of Hamilton-Fletcher et al. (2018) for SSAh conditions.** Violin plot of mean SSAh responses in the early blind (EB, $N = 32$), late blind (LB, $N = 23$), and typically sighted control (TS, $N = 63$) groups with kernel density estimated with Gaussian kernels. Individual data points are plotted with jitter. From the data of Hamilton-Fletcher et al. (2018), we excluded 4 LB participants whose blindness onsets were before the age of 12 years. A value of 1 indicates a fully congruent SSAh (*Kiki* matched with an angular shape and *Bouba* with a round shape in all four trials), and a value of 0 indicates a fully incongruent SSAh (*Kiki* matched with a round shape and *Bouba* with an angular shape in all four trials). The red point indicates mean group response and the error bars indicate 95% confidence interval of the mean obtained by smoothed bootstrapping with a Gaussian kernel. The dotted line indicates chance level performance. The EB group but not the LB group statistically significantly differed from the TS group in their SSAh responses. Moreover, while the LB and the TS group exhibited systematic SSAh responses (red asterisks), the EB group did not, performing no different from what would be expected by chance. (*: $0.01 \leq p < 0.05$; **: $0.001 \leq p < 0.01$; ***: $0 \leq p < 0.001$).

A second regression model aimed to find evidence for a systematic SSAh in each group indicated that while the TS and the LB groups showed a robust SSAh, statistically significantly different from what would be expected by chance alone ($\beta_{TS} = 4.393$, $SE = 0.994$, $p < 0.001$, 95% *CI* [2.778, 6.640]; $\beta_{LB} = 2.519$, $SE = 1.206$, $p = 0.037$, 95% *CI* [0.425, 5.539]), the EB group did not ($\beta_{EB} = 0.1630$, $SE = 0.8420$, $p = 0.847$, 95% *CI* [-1.584, 2.055]).

Regressing the data from the LB participants in the present study with a generalized linear mixed model with binomial link, participant ID as a random intercept factor, and the duration of degraded vision (= Current age − Age at onset of degraded vision) as fixed factor, we found no evidence that duration of degraded vision in LB individuals influenced SSAh (β = 0.042, *SE* = 0.147, *p* = 0.777, 95% *CI* [-0.330, 0.659]). Repeating the analysis with duration of blindness as the fixed factor also failed to reveal any effect of blindness duration on the SSAh in the LB group (β = 0.008, *SE* = 0.138, *p* = 0.956, 95% *CI* [-0.473, 0.704]).

Reanalyzing the LB data from Hamilton-Fletcher et al. (2018) in the SSAh condition with the same model after excluding LB participants whose age at blindness was below 12 years once again failed to reveal a significant effect of blindness duration, either expressed in years (β = 0.117, *SE* = 0.107, *p* = 0.272, 95% *CI* [-0.120, 0.423]) or as a percentage of life (β = 0.058, *SE* = 0.063, *p* = 0.351, 95% *CI* [-0.088, 0.230]).

S4. Comparing the Non-Indian TS Group to the Indian TS Group

We compared the response of the non-Indian TS individuals (*N* = 42) to that of Indian TS individuals (*N* = 28) employing the same models as detailed in *S2. Detailed Descriptions of Statistical Analyses* (See Fig. S4). There were only two groups for these analyses: non-Indian TS, and Indian TS. For both SSAh and SSAv responses, we first ascertained whether the non-Indian TS group differed from the Indian TS group. For this purpose, the Indian TS group was set as the reference group. Thereupon we investigated whether each group displayed an above chance-level SSA by means of a zero-intercept regression model. For the SSAh analysis, a generalized linear mixed model was used with *Group* as the fixed factor and *Participant ID* as a random intercept factor. The SSAv analysis only had one fixed factor, *Group,* since a single trial per participant obviated the need for a random effect term.

In both SSAh and SSAv contexts, the non-Indian TS group did not perform statistically significantly differently from the Indian TS group (SSAh: $\beta_{\Delta\text{TS (Non-Indian)}}$ = 0.443,

SE = 2.027, *p* = 0.827, 95% *CI* [-5.025, 5.720], SSAv: $\beta_{\Delta TS\ (Non\text{-}Indian)}$ = 0.725, *SE* = 0.721, *p* = 0.314, 95% *CI* [-0.697, 2.209]). Additionally, both groups showed robust SSA responses (SSAh: $\beta_{TS\ (Indian)}$ = 9.738, *SE* = 1.855, $p < 0.001$, 95% *CI* [6.691, 15.807], $\beta_{TS\ (Non\text{-}Indian)}$ = 10.181, *SE* = 1.789, $p < 0.001$, 95% *CI* [6.584, 16.046]; SSAv: $\beta_{TS\ (Indian)}$ = 1.5261, *SE* = 0.493, *p* = 0.002, 95% *CI* [0.640, 2.617], $\beta_{TS\ (Non\text{-}Indian)}$ = 2.251, *SE* = 0.5256, $p < 0.001$, 95% *CI* [1.340, 3.456]).

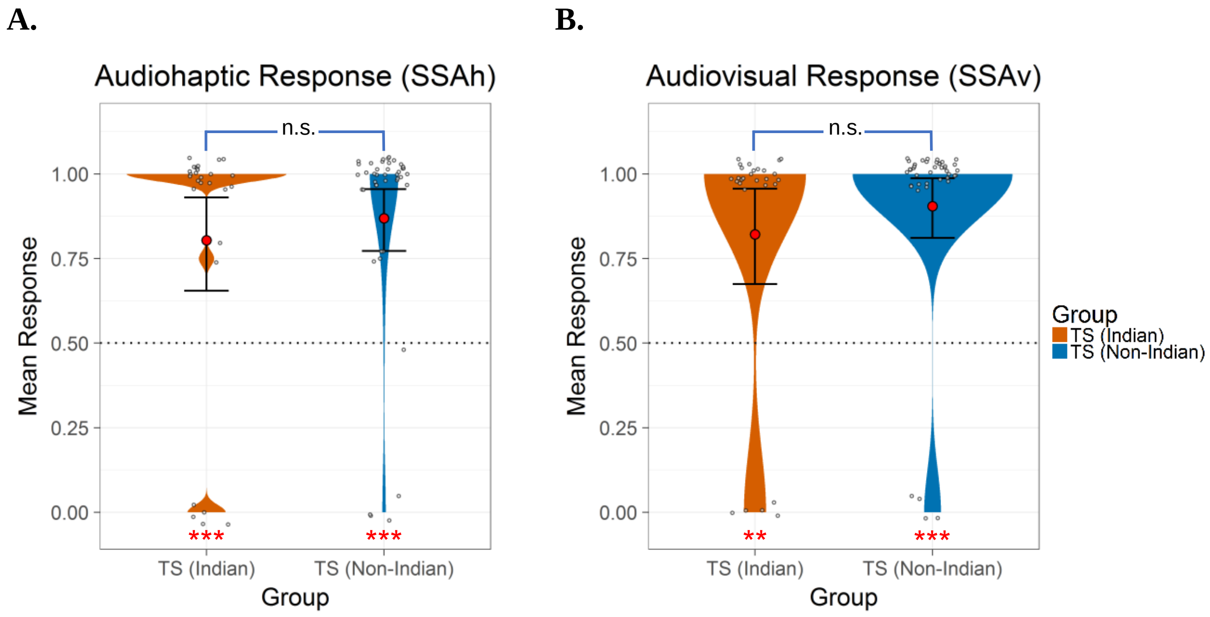

Figure S4. **Mean responses in the audiohaptic and audiovisual conditions in the Indian and non-Indian sighted control (TS) subgroups.** (A) Violin plot of Mean audiohaptic (SSAh) response with kernel density estimated with Gaussian kernels. Individual data points are shown with jitter. A value of 1 indicates a fully congruent SSAh (*Kiki* matched with an angular shape and *Bouba* with a round shape in all four trials), and a value of 0 indicates an incongruent SSAh (*Kiki* matched with a round shape and *Bouba* with an angular shape in all four trials). The red points indicate mean group responses and the error bars indicate 95% confidence interval of the mean obtained by smooth bootstrap with a Gaussian kernel; the dotted line indicates chance level performance. Both the Indian and the non-Indian TS groups showed a robust SSAh (red asterisks), and the non-Indian TS group was not found to be statistically significantly different from the Indian TS group. (B) Mean audiovisual response using similar convention as in (A). Both the non-Indian and the Indian TS groups showed a robust SSAv and the non-Indian TS group did not significantly differ from the Indian TS group (*: $0.01 \leq p < 0.05$; **: $0.001 \leq p < 0.01$; ***: $0 \leq p < 0.001$).

References

Bates, D., Mächler, M., Bolker, B., & Walker, S. (2015). Fitting linear mixed-effects models using lme4. *Journal of Statistical Software*, *67*(1), 1–48.

Halekoh, U., & Højsgaard, S. (2014). A Kenward-Roger approximation and parametric bootstrap methods for tests in linear mixed models – The R package pbkrtest. *Journal of*

Statistical Software, *59*(9), 1–30.

Hamilton-Fletcher, G., Pisanski, K., Reby, D., Stefańczyk, M., Ward, J., & Sorokowska, A. (2018). The role of visual experience in the emergence of cross-modal correspondences. *Cognition*, *175*, 114–121.

Hardy, M. A. (1993). *Regression with dummy variables* (Vol. 93). Newbury Park, CA: Sage.

R Core Team. (2016). R: A language and environment for statistical computing. Vienna, Austria. Retrieved from https://www.r-project.org/

Wolodzko, T. (2018). Kernelboot: Smoothed bootstrap and random generation from kernel densities. Retrieved from https://cran.r-project.org/package=kernelboot
